# Patients return to sports and to work after successful treatment of septic arthritis following anterior cruciate ligament reconstruction

**DOI:** 10.1007/s00167-021-06819-x

**Published:** 2021-12-06

**Authors:** Alexander Themessl, Felix Mayr, Kate Hatter, Marco-Christopher Rupp, Jonas Pogorzelski, Andreas B. Imhoff, Stefan Buchmann

**Affiliations:** 1 Department of Orthopaedic Sports Medicine, Technical University of Munich, Ismaninger Str. 22, 81675 Munich, Germany; 2 Department of Surgery/Emergency Medicine, Duke University Hospital, Durham, USA; 3 Orthopaedisches Fachzentrum (OFZ) Weilheim/Garmisch/Starnberg/Penzberg, Weilheim, Germany

**Keywords:** Septic arthritis, Anterior cruciate ligament reconstruction, Return to sport, Return to work, Complication, Revision

## Abstract

**Purpose:**

To determine specific return to sports (RTS) and return to work (RTW) rates of patients with septic arthritis following anterior cruciate ligament reconstruction (ACLR), and to assess for factors associated with a diminished postoperative return to physical activity after successful eradication of the infection.

**Methods:**

In this study, patients who were treated for postoperative septic arthritis of the knee following anterior cruciate ligament reconstruction between 2006 and 2018 were evaluated at a minimum follow-up (FU) of 2 years. Patients’ outcomes were retrospectively analyzed using standardized patient-reported outcome scores including the Lysholm score and the subjective IKDC score, as well as return to sports and return to work questionnaires to assess for the types, number, and frequency of sports performed pre- and postoperatively and to evaluate for potential occupational changes due to septic arthritis following ACLR. To assess for the signifiance of the graft at follow-up, outcomes were compared between patients with a functioning graft at FU and those without, as well as between patients with initial graft retention and those with graft removal and consecutive revision ACLR.

**Results:**

Out of 44 patients eligible for inclusion, 38 (86%) patients at a mean age of 36.2 ± 10.3 years were enrolled in this study. At a mean follow-up of 60.3 ± 39.9 months, the Lysholm score and the subjective IKDC score reached 80.0 ± 15.1 and 78.2 ± 16.6 points, respectively. The presence of a graft at FU yielded statistically superior results only on the IKDC score (*p* = 0.014). There were no statistically significant differences on the Lysholm score (n.s.) or on the IKDC score (n.s.) between patients with initial graft retention and those with initial removal who had undergone revision ACLR. All of the included 38 patients were able to return to sports at a median time of 8 (6–16) months after their last surgical intervention. Among patients who performed pivoting sports prior to their injury, 23 (62.2%) returned to at least one pivoting sport postoperatively. Overall, ten patients (26.3%) returned to all their previous sports at their previous frequency. The presence of a graft at FU resulted in a significantly higher RTS rate (*p* = 0.010). Comparing patients with initial graft retention and those with graft removal and consecutive revision ACLR, there was no statistically significant difference concerning the RTS rate (n.s.). Thirty-one patients (83.8%) were able to return to their previous work.

**Conclusion:**

Successful eradication of septic arthritis following anterior cruciate ligament reconstruction allows for a postoperative return to sports and a return to work particularly among patients with ACL-sufficient knees. However, the patients’ expectations should be managed carefully, as overall return rates at the pre-injury frequency are relatively low.

**Level of evidence:**

IV.

**Supplementary Information:**

The online version contains supplementary material available at 10.1007/s00167-021-06819-x.

## Introduction

In recent years, the understanding of correctly diagnosing and treating septic arthritis (SA) of the knee following anterior cruciate ligament reconstruction (ACLR) has steadily increased [6, 7, 23, 27, 28, 35, 41, 43]. Particularly, graft retaining protocols emphasizing the combination of antibiotic regimen and surgical management have led to favorable postoperative objective results and satisfying patient-reported outcomes [10, 16, 21, 34, 47]. But while many conventional patient-reported outcome scores as well as laxity measurements have demonstrated their importance, particularly in a scientific setting, such assessments are often abstract at the individual level [3]. Meanwhile important personal aspects for patients, such as a successful athletic and occupational rehabilitation after this severe complication, have gained little attention in the previous literature [8, 29, 32]. Yet as patients affected by septic arthritis following anterior cruciate ligament reconstruction tend to be young and active, more thorough information about prognoses of postoperative activity levels as well as impacts on the professional lives is crucial. Therefore, the purpose of this study was to evaluate the return to sports (RTS) and return to work (RTW) rates after septic arthritis following ACLR at a medium- to long-term follow-up and to assess for factors associated with diminished postoperative return to sports and return to work rates after successful eradication of septic arthritis. Our primary hypothesis was that patients with a graft sufficient knee at follow-up would be able to return to sports and work at higher rates compared to patients with graft insufficient knees. Furthermore, it was hypothesized that among patients with a graft sufficient knee at follow-up, initial graft retention would prove beneficial with respect to clinical outcomes compared to initial graft removal and consecutive ACLR revision.

## Materials and methods

A monocentric Institutional Review Board approved Level IV retrospective case series was conducted (institution blinded for review, permit number 279/20 S-SR) and written informed consent was obtained from each patient before participation. Patients who underwent open or arthroscopic treatment for septic arthritis following anterior cruciate ligament reconstruction at the main author’s institution between 01/2006 and 12/2018 with a minimum follow-up of 24 months after their last treatment of septic arthritis were eligible for this study. Patients were included both in cases of graft retention and graft removal during the treatment of their septic arthritis. Patients were excluded if they were non-residents or if they had undergone concomitant (osteo)chondral transplantation, mechanical alignment correction, or multi-ligament surgery during index ACLR. Eligible patients were contacted via mail and were considered lost to follow-up if they refused to participate or failed to respond to the mailed survey. Baseline demographics (e.g., sex, age, operated side) were gathered from medical records and operative information on the type of index ACLR (primary vs. revision), concomitant procedures during index surgery, graft type, operative technique (single bundle vs. double bundle), staging according to Gaechter classification [50], and microorganisms causative for infection were obtained from the clinic records for all patients. The procedure (primary or revision ACLR) which preceded the onset of septic arthritis was considered as the index ACLR.

### Diagnosis and treatment of postoperative septic arthritis

The diagnostic algorithm and treatment regimen applied for suspected cases of septic arthritis has previously been published by this group [37]. In summary, the diagnosis of septic arthritis after ACLR was obtained based on clinical manifestations (e.g., pain, joint swelling, local erythema, hyperthermia, and purulent secretion), laboratory results (e.g., C-reactive protein > 0.5 mg/dl, white blood cell count > 10,000/ µl), and microbiologic findings from a joint aspirate or tissue gathered during arthroscopy. Grading was based on arthroscopic findings according to the Gaechter classification [50].

The standard of care for treatment of septic arthritis following ACLR includes a combination of systemic antibiotics in addition to surgical irrigation and debridement. A calculated antibiotic treatment regime is initiated and adapted based on pathogen sensitivity testing and the patient’s prior history of antibiotic resistance. Usually, an initial intravenous therapy for 10–14 days followed by a period of at least four weeks of oral antibiotics is utilized and thus was used in this study. Arthroscopic irrigation and debridement (I&D) was performed in every suspected or confirmed case of septic arthritis, and performed repeatedly, if necessary, until negativity of microbiological cultures was sustained after 48 h. Conversion to an open revision was reserved for severe cases of septic arthritis (Gaechter IV) or in cases of unsuccessful arthroscopic I&D. Grafts were salvaged unless they showed signs of insufficiency in arthroscopic testing or osteomyelitis involving the bone tunnels used for anterior cruciate ligament (ACL) fixation, as well as in cases of persistent infection despite repeated I&D. Suction drains were placed intraoperatively and removed after drainage had ceased, usually around the third postoperative day. Patients were restricted to bed rest for 48 h postoperatively, with consecutive partial weight bearing for the first two weeks after treatment. Eradication of septic arthritis was considered successful after a complete cease of clinical and laboratory signs of infection including negativity of all microbiologic cultures.

### Outcome measures

Clinical outcomes were evaluated at a minimum of two years after the last surgical treatment for septic arthritis and included the Lysholm score and the subjective knee evaluation form administered by the International Knee Documentation Committee (IKDC). To assess for a minimal clinically important difference (MCID), a difference of 10 points and of 9 points were used for the Lysholm score and the IKDC scores, respectively [31]. Additionally, a return to sports and return to work questionnaire was developed, as no validated return to sports questionnaire exists for septic arthritis following ACLR. This form was based on questionnaires used in previous studies about postoperative sporting activities and adjusted for our study population [14]. The questionnaire was divided into three parts. The first part contained information about the patients’ medical and surgical history before and after the treatment of septic arthritis at our institution, overall postoperative satisfaction (very satisfied, partially satisfied, barely satisfied, and not satisfied), and persisting complaints in the ipsilateral knee (instability, pain, stiffness, other). The second part of the questionnaire assessed types, number, and frequency of sports or activities carried out for at least six months prior to the injury leading to the index surgery, as well as types, number, and frequency at the time of follow-up. Types of sports were categorized dichotomously into pivoting sports and non-pivoting sports [18]. Frequency was categorized as low (a maximum of 30–60 min per week), medium (up to 60 min at least 2–3 times per week), and high (at least 60 min and greater than 3 times per week). Furthermore, the time to full return to sports, postoperative reduction of frequency, or the discontinuation of any sports previously performed was evaluated. Return to sports was defined as the postoperative performance of any type of sports carried out at least 30–60 min per week. Return to the previous level of sports was considered to be the continuation of all sports at the same frequency as before the index surgery. Reasons for discontinuation of any sports or for the reduction of frequency of sports were further evaluated (ipsilateral knee instability, pain, stiffness, fear of re-injury, other). The third part of the questionnaire collected information on occupational behaviors and employment. The level of employment was assessed according to various activities carried out during work (e.g., sitting, standing, lifting, and carrying) [13, 45]. These levels were then categorized dichotomously into sedentary labor and non-sedentary/physical labor based on the intensity and duration of the work activities.

To assess for the significance of a graft and its characteristics (initial graft salvage vs. initial graft removal with or without consecutive revision ACLR), follow-up outcome scores and return rates were compared between patients with graft sufficient knees (presence of a graft at follow-up) and those without (no graft at follow-up), as well as between patients with initial graft retention and those with initial graft removal and consecutive ACLR revision. Further variables including age (> 30 years vs. < 30 years), sex, surgical technique used for index ACLR, revision status for index ACLR, concomitant injuries, and microorganisms causative for infection were analyzed regarding their effect on postoperative outcomes.

### Statistical analysis

Statistical analysis for this study was performed using the SPSS Software version 26 (IBM, statistics). Distribution of the variables was assessed using the Kolmogorov–Smirnov test. Continuous non-parametric variables are presented as median (1st quartile–3rd quartile) and parametric data are presented as mean ± standard deviation. Absolute numbers and percentages were used for categorial variables. The Mann–Whitney *U* test was applied for comparison of continuous non-parametric values and the Chi-square test was used for comparison of categorial variables. All *p* values were calculated two-tailed and the level for statistical significance was set at *p* < 0.05. To assess for the statistical power of this study, a post hoc power analysis was performed for outcome comparison of the IKDC score between patients with graft removal and graft retention using two-tailed tests. It was shown that the required sample size of 38 could achieve an adequate power of 0.90 with an alpha of 0.05 using G*Power (version 3.1.9, Düsseldorf) [11].

## Results

### Baseline demographics

Between 2006 and 2018, 57 patients underwent surgical treatment for septic arthritis following ACLR at a single institution. Thirteen patients were excluded due to a nonresident status or because of reconstructive concomitant procedures. Six (14%) of the remaining 44 patients eligible for inclusion were lost to follow-up. Thus, a total of 38 patients (86%) were included in the final analysis. Demographic and surgical data of the included patients are presented in Table 1.

**Table 1:** Patient characteristics of the study group

Demographics	Value
Age at diagnosis of septic arthritis, years	28.3 ± 10.0
Age at follow-up, years	36.2 ± 10.3
Sex	
Male Female	29 (76.3) 9 (23.7)
Affected side Left Right	15 (39.5) 23 (60.5)
Technique of index ACLR (HS autograft)	
SB DB No information	21 (55.3) 15 (39.5) 2 (5.3)
Index ACLR	
Primary Revision	26 (68.4) 12 (31.6)
Concomitant procedures^a^	
Medial meniscus	5 (13.2)
Lateral meniscus	7 (18.4)
Graft removal during eradication of infection	
Yes Revision ACLR until FU No revision ACLR until FU No	13 (34.2) 6 (46.1) 7 (53.9) 25 (65.8)
Microbiologic findings	
Coagulase negative staphylococci *Staphylococcus aureus* Others^b^ None	26 (66.7) 6 (15.4) 5 (12.8) 2 (5.1)
Further surgeries^c^ ACLR Ipsilateral re-rupture Contralateral rupture Ipsilateral extra-articular stabilization Ipsilateral arthrolysis Ipsilateral arthroplasty	4 (10.5) 2 (50.0) 2 (50.0) 1 (2.6) 2 (5.3) 1 (2.6)

Continuous data are presented as mean ± standard deviation (SD) unless otherwise noted. Categorical data are presented as *n* (%)

*FU* follow-up, *ACLR* anterior cruciate ligament reconstruction, *HS* Hamstring, *SB* single bundle, *DB* double bundle

^a^Partial meniscectomy or suture during the index surgery

^b^e.g. *Cutibacterium acnes*, streptococci and *Corynebacterium*

^c^All (revision) surgeries that patients underwent at the index or contralateral knee between successful eradication of septic arthritis and follow-up

### Patient-reported outcomes

Follow-up scores were available for all 38 patients. The total Lysholm score and subjective IKDC score of the included patients reached a mean of 80.0 ± 15.1 and 78.2 ± 16.6 points after 60.3 ± 39.9 months. Overall, 32 patients (84.2%) were either very satisfied (*n* = 18; 47.4%) or satisfied (*n* = 14; 36.8%) with their postoperative results. Six patients (15.8%) were either barely satisfied (*n* = 3; 7.9%) or not satisfied (*n* = 3; 7.9%). Postoperative patient satisfaction was associated with superior results both on the Lysholm score (*p* = 0.007) and the subjective IKDC score (*p* = 0.002). Subgroup comparisons of the outcome scores with respect to graft characteristics are listed in Table 2.

**Table 2:** Subgroup comparison of PROMs

Parameters	Lysholm score	*p* value	IKDC score	*p* value
Primary index ACLR		0.025		n.s
Yes (*n* = 26) No (*n* = 12)	84.9 ± 12.0 72.1 ± 17.8		81.9 ± 13.5 69.9 ± 20.5	
Graft at FU		n.s		0.014
Yes (*n* = 31) No (*n* = 7)	82.8 ± 14.7 72.1 ± 14.9		81.4 ± 15.5 62.5 ± 13.1	
Graft characteristic at FU^a^		n.s		n.s
Retention (*n* = 25) rACLR (*n* = 6)	85.2 ± 13.5 72.8 ± 16.5		83.7 ± 13.9 72.1 ± 19.5	

Outcome scores of patients with regard to graft characteristics. Scores are presented as mean ± standard deviation, the *p* value considered significant was (*p* < 0.05)

*PROMs* patient-reported outcome measures, *FU* follow-up, *IKDC* subjective form of the international knee documentation committee, *rACLR* revision anterior cruciate ligament reconstruction

^a^patients with a graft at follow-up (*n* = 31) with either initial graft retention or initial graft removal and consecutive rACLR

Patients’ age and sex, the surgical technique used for index ACLR (single bundle vs. double bundle), the presence of concomitant injuries, or the microorganisms found (coagulase negative staphylococci vs. *Staphylococcus aureus*) did not significantly affect the Lysholm score (n.s.) or the IKDC score (n.s.) at follow-up. Thirty-one patients (81.6%) reported persisting complaints regarding the operated knee. Pain was most frequently reported (*n* = 20; 52.6%), followed by a decreased range of motion and instability (*n* = 13; 34.2%). Seven patients (18.4%) reported no persisting issues at final follow-up.

### Postoperative return to sports

All 38 patients included in this study reported to have participated in sports on a regular basis within six months of their index ACL injury. Prior to their injuries, two patients (5.4%) reported to have engaged in sports at a low frequency, twenty-three patients (62.2%) at a medium frequency, and eleven patients (28.9%) at a high frequency.

**Fig. 1:**
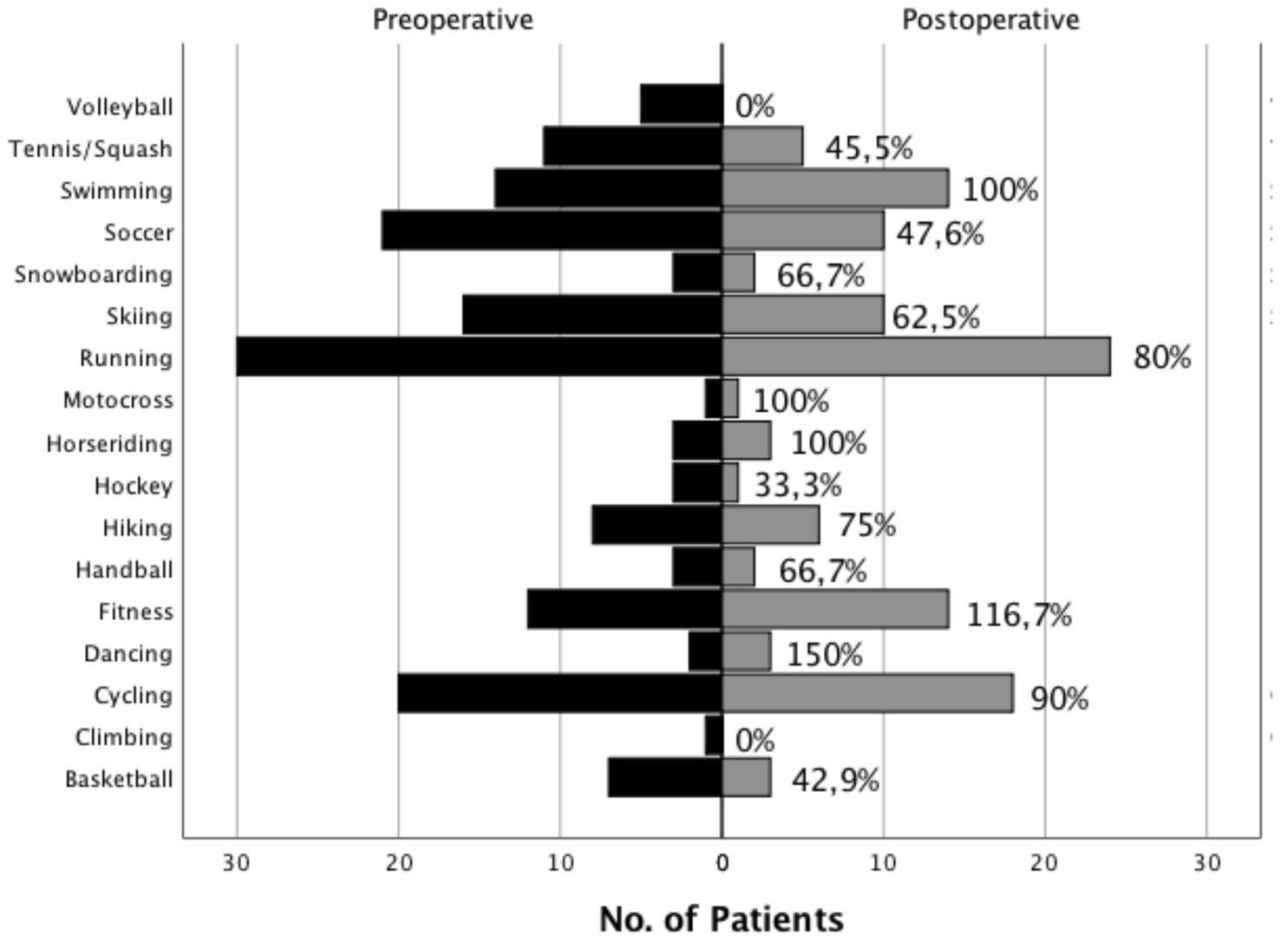
Activities and sports practiced by the patients of this study preoperatively (prior to index ACL injury) and postoperatively at the time of follow-up. Direct return rates for each sport are listed within the figure, the absolute number of patients is provided on the *x*-axis

At a mean follow-up of 99.3 ± 29.7 months, all patients reported to have returned to sports at a median duration of 8 months (6–16 months) after their last treatment for septic arthritis. Twenty-one patients (55.3%) had discontinued at least one sport because of their injured knee. Figure 1 displays the activities and rates of the sports performed pre- and postoperatively.

Of the sports discontinued, all sports were pivoting sports (Table 3). Eighteen patients (51.4%) reported a reduction of frequency compared to their preinjury status. Overall, ten patients (26.3%) reported to have returned to all their preinjury sports at their preinjury frequency at follow-up. Reasons for discontinuation of any sport are listed in Table 4.

**Table 3:** Sub-analysis of return to pivoting sports

RTS characteristics	Value
Preoperative participation in pivoting sports Return to any pivoting sport^a^ Return to all pivoting sports^b^ Return to all pivoting sports at previous frequency^c^	37 (97.4) 23 (62.2) 16 (43.2) 10 (27.0)

Data are presented as *n* (%)

*RTS* return to sports

^a^Post-operative return to any one pivoting sport performed preoperatively

^b^Post-operative return to all pivoting sports performed preoperatively

^c^Post-operative return to all pivoting sports performed preoperatively at the preoperative frequency

**Table 4:** Reason for discontinuation of sports

Issues	Total number of reasons (*n* = 32)	Number of single reasons (*n* = 13)
Instability *n*, (%) Pain *n*, (%) Fear of re-injury *n*, (%) Others *n*, (%)	13 (40.6) 14 (43.8) 17 (53.1) 7 (21.9)	2 (15.4) 2 (15.4) 6 (46.2) 3 (23.1)

Listing of the reasons given for discontinuation of at least one sport including multiple answers (total number of reasons) and single answers (number of single reasons)

The only factor significantly associated with diminished postoperative return to sports was the lack of a graft at final follow-up (*p* = 0.010), while the graft characteristic (initial graft salvage vs. initial graft removal with consecutive ACLR revision) did not significantly affect return to sports rates (n.s.). A detailed list of the main activities specific subgroups of patients returned to is provided in the Supplementary Appendix.

### Postoperative return to work

Return to work data was available for 37 patients. All patients were either employed or students at an educational institution at time of diagnosis of their septic arthritis. Overall, twenty patients (54.1%) had a sedentary occupation, and seventeen patients (45.9%) had a non-sedentary/physical occupation. Six of the patients (16.2%) had to either transfer to lower the intensity work within their job or change jobs entirely due to the implications of their injury, while thirty-one (83.8%) continued at the same level of intensity within their previous occupation. Of the six patients who had to change jobs or transfer within their workplace, four (66.7%) carried out a non-sedentary/physical occupation preoperatively and two patients (33.3%) worked in a sedentary job. There was no association between the ability of patients to return to sports and to return to work (n.s.).

## Discussion

The main finding of this study was that successful treatment of septic arthritis after ACLR enabled patients to return to sports, even though return rates to all sports at the previous frequency were diminished. Furthermore, all patients returned to work and most of them returned to their pre-injury occupation. In accordance with our primary hypothesis, the presence of a graft at follow-up was advantageous for return to sports. Yet, this was true regardless of the graft characteristic (initial salvage vs. graft removal with consecutive revision ACLR), thus rejecting our secondary hypothesis.

To our knowledge, this is the largest study on postoperative patient outcomes after septic arthritis following ACLR to date and the first one to evaluate the return to sports and return to work rates of these patients in detail. In our study, all 38 patients successfully returned to any sports after treatment of septic arthritis, and of the 37 patients who had performed pivoting sports prior to their injury, almost two-thirds (62.2%) restarted a pivoting sport postoperatively. There was a trend for a higher return to pivoting sports among patients under the age of 30, but regardless of age, highest return to sport rates were seen in typical endurance and low demand activities with little risk of (re-)injury (e.g., swimming, running, and cycling). The overall return rate to all previous sports at the pre-injury frequency was relatively low at 26.3%.

Recent advances in the prevention of postoperative septic arthritis after ACLR, namely different methods of perioperative antibiotic graft soaking, have raised hopes to efficiently reduce infection rates, likely without relevant adverse effects on the graft [4, 12, 24, 30, 33, 36, 42, 51]. Nonetheless, concerns remain regarding this practice among surgeons, and septic arthritis continues to pose a threat to patients’ outcomes [47, 52]. Besides traditional outcome measures including patient-oriented outcome forms and laxity testing, participation-based parameters such as return to sports and return to work can help to clarify the impact of this condition on patients’ lives and assist in guiding postoperative expectations [38, 44, 46]. But in the current literature, return to sports rates after SA following ACLR are heterogeneous, ranging between 50 and 100%, often lacking specification regarding the types or levels of sports performed, and therefore render detailed advice difficult [1, 5, 8, 22, 28].

In two case–control studies, Abdel-Azis et al. [1] and Bostrom Windhamre et al. [7], found a return to (recreational) activities in 56% and 62.5% of their patients without further stating the types or characteristics of the sports. Binnet et al. [5] reported a return to previous level in competitive sports in one out of three patients, but with a limited sample size (*n* = 6). Similar to our findings, Calvo et al. [8] and Monaco et al. [29] reported a full return to athletic activity in their studies in all seven and all 14 patients, respectively. However, the authors describe higher return rates to a preinjury level of activity compared to our study (85.7% and 78.6%, respectively). Interestingly, the authors point out the importance of initial graft retention for a return to sports. While there is evidence that initial graft removal is associated with inferior postoperative subjective and objective outcomes (particularly without consecutive ACLR revision) [15, 37], we were not able to find an association between initial graft removal and lower return to sports rates. This is in concordance with findings by Waterman et al.[48], who found no correlation between initial graft removal during the treatment of SA and a postoperative return to active military duty. In our study, persistent ACL deficiency at follow-up was the only patient-dependent factor significantly associated with an inferior RTS rate, which is consistent with the findings of previous studies, showing an association of ACL deficiency after treatment for SA and diminished Tegner activity scores [15, 41]. Patients in this study, who underwent initial graft removal and consecutive ACLR revision reached similar RTS rates as patients with initial graft retention. Therefore, it is likely that it is the presence of a graft that matters, not whether it is a primary or revision graft, even though negative implication on return rates may remain in either case. But implications on return to sports rates are also seen in patients who undergo ACLR without postoperative septic arthritis [3, 53]. In their systematic review, Ardern et al.[3] found a mean return to any type of sports of 82%, and a return to previous level of activity of 63% after ACLR at 44 months. In a later case series, the same authors reported a return to preinjury level of sports of 44.6% after a mean of 39 months[2]. Similar results are reported by Grassi et al.[17], who in their systematic review on postoperative outcomes after revision ACLR report a RTS rate of 85%, and a return to preinjury level of sports of 53% after 4.2 years.

As to why patients do not return to sports, patient-associated factors, such as gender and the level of sports performed preoperatively, have to be considered as well as structural and functional deficits of the injured knee [39, 40], which is highlighted by the fact, that about 80% of the patients in this study reported persisting issues with their operated knee. But interestingly, only about half of the patients who reported to have stopped any sports in this study did so because of the operated knee, indicating that additional aspects may be of relevance. There is increasing awareness of the impact of psychological factors such as a lack of motivation, fear of re-injury, or a shift of interests on patients’ capability to return to activity postoperatively [9, 15, 25, 28, 41]. The effect of these so-called ‘soft factors’ might also explain the discrepancies often found between solid clinical outcome parameters and diminished return to sports rates found in our study and in the literature [13, 14, 20, 49]. For example, in our study, fear of re-injury was the most frequently given answer for the discontinuation of a sport. While this aspect has previously been highlighted regarding patients after ACLR without postoperative complications, the repercussions may be of even greater relevance in patients who have suffered from a condition as complicated and disabling as septic arthritis [3, 26, 38].

Despite the high relevance for patients to be able to work after a surgery, particularly among young patients, there remains a scarcity of studies investigating postoperative return to work rates. In our study, all patients were able to return to work after successful eradication of infection. As expected, patients with physical demanding occupations were affected more often as evidenced by a change of job or transfer to a lower intensity work within their job. This finding is intuitive and consistent with results presented by Groot et al. [19] who describe a return to work rate of 92% (*n* = 82) after ACLR, with the highest rates of return to employment among patients in initially sedentary jobs.

While this study does demonstrate important findings, it is not without limitations. From a methodological point of view, we point out that this was a retrospective monocentric study. Therefore, the lack of randomization inherent to this study design bears an increased risk for selection bias. However, regarding demographic and surgical aspects, the cohort of this study well reflects collectives described in the literature [28]. Also, the RTS questionnaire used in this study was a non-standardized questionnaire with a potential question order bias. Yet, our questionnaire was based on previously published protocols to attempt to standardize and reduce the risk of bias [13, 14]. Finally, retrospective studies involve a risk for recall bias. But the key parameters assessed in this study (return to sports and return to work) are ongoing variables, ideally still being true at the time of the follow-up and thus reducing the risk for recall bias with respect to these key parameters. Also, as patient outcomes were assessed in an intention to treat fashion, revision surgeries after eradication of septic arthritis might have confounded the results. However, as these revision surgeries were few and as we intended to give an adequate representation of all patients with septic arthritis following ACLR, including the potential revisions, all patients were included for final analysis.

Finally, while septic arthritis following ACLR remains an infrequent complication, it may come with repercussions on patients’ physical activity and professional life. While no clear activity recommendations can be drawn from our data, this study is intended to raise awareness of the factors associated with diminished return to sports and work rates and to help clinical practitioners guide their patients’ expectations in these situations.

## Conclusion

Patients who undergo successful treatment of septic arthritis following anterior cruciate ligament reconstruction can return to both low and to high demand sports, with highest return rates seen for typical endurance sports. Yet, a return to preinjury sports at a preinjury level among these patients is relatively low. ACL deficiency at follow-up is predictive of inferior subjective outcome scores and leads to a decreased likelihood of returning to preinjury sports. Finally, successful eradication of septic arthritis allows for a substantial return to previous work.

## Supplementary Information

Below is the link to the electronic supplementary material.

Supplementary file1 (DOCX 18 KB)

## Abbreviations

SA: Septic arthritis
ACLR: Anterior cruciate ligament reconstruction
RTS: Return to sports
I&Ds: Irrigations and debridements
ACL: Anterior cruciate ligament
IKDC: International Knee Documentation Committee
MCID: Minimal clinically important difference
HS: Hamstring
SB: Single bundle
DB: Double bundle
PROMs: Patient-reported outcome measures
