# Supplementary material for: Patients return to sports and to work after successful treatment of septic arthritis following anterior cruciate ligament reconstruction
Source: Knee Surg Sports Traumatol Arthrosc. 2021 Dec 6;30(6):1871–9. doi: 10.1007/s00167-021-06819-x (PMC9159288; doi:10.1007/s00167-021-06819-x)
Supplement: Supplementary file 1 — Supplementary file1 (DOCX 18 KB) [file 167_2021_6819_MOESM1_ESM.docx]

| Top Five Activities of Patients Before and After Surgery (n = 38) ^a^ | | | | | |
| --- | --- | --- | --- | --- | --- |
|  |  | |  | |  |
|  | Preoperative | | Postoperative | |  |
|  | No. of Patients | Percentage (%) | No. of Patients | Percentage (%) | Direct Rate of Return (%) |
| Top sports, women | | | | | |
| Running | 7 | 77.8 | 6 | 66.7 | 85.7 |
| Ski | 6 | 66.7 | 5 | 55.5 | 83.3 |
| Cycling | 5 | 55.6 | 3 | 33.3 | 60 |
| Tennis/Squash | 2 | 22.2 | 1 | 11.1 | 50 |
| Soccer | 2 | 22.2 | 1 | 11.1 | 50 |
| Top sports, men |  |  |  |  |  |
| Running | 21 | 72.4 | 17 | 58.6 | 80.9 |
| Soccer | 18 | 62.1 | 8 | 27.6 | 44.4 |
| Cycling | 15 | 51.7 | 14 | 48.3 | 93.3 |
| Ski | 9 | 31.0 | 5 | 17.2 | 55.6 |
| Tennis/Squash | 8 | 27.6 | 4 | 13.8 | 50 |
| Top sports, older patients (>30 y)^b^ | | | | | |
| Running | 9 | 75.0 | 4 | 33.3 | 44.4 |
| Cycling | 7 | 58.3 | 6 | 50 | 85.7 |
| Tennis/Squash | 5 | 41.7 | 2 | 16.7 | 40 |
| Ski | 4 | 33.3 | 2 | 16.7 | 50 |
| Soccer | 4 | 33.3 | 1 | 8.3 | 25 |
| Top sports, younger patients (<30 y)^b^ | | | | | |
| Running | 19 | 73.1 | 19 | 73.1 | 100 |
| Soccer | 16 | 61.5 | 8 | 30.8 | 50 |
| Cycling | 13 | 50 | 11 | 42.3 | 84.6 |
| Ski | 11 | 42.3 | 8 | 30.8 | 72.7 |
| Tennis/Squash | 5 | 19.2 | 3 | 11.5 | 60 |
| Top sports, graft salvage | | | | | |
| Running | 18 | 72 | 15 | 60 | 83.3 |
| Soccer | 15 | 60 | 8 | 32 | 53.3 |
| Cycling | 14 | 56 | 12 | 48 | 85.7 |
| Ski | 11 | 44 | 7 | 28 | 63.6 |
| Tennis/Squash | 6 | 24 | 4 | 16 | 66.7 |
| Top sports, graft removal (+ACLR)^c^ | | | | | |
| Running | 5 | 83.3 | 5 | 83.3 | 100 |
| Ski | 3 | 50.0 | 3 | 50.0 | 100 |
| Cycling | 2 | 33.3 | 1 | 16.7 | 50 |
| Tennis/Squash | 1 | 16.7 | 1 | 16.7 | 100 |
| Soccer | 1 | 16.1 | 1 | 16.1 | 100 |
| Top sports, graft removal (- ACLR)^c^ | | | | | |
| Running | 5 | 71.4 | 3 | 42.9 | 60 |
| Cycling | 4 | 57.1 | 4 | 57.1 | 100 |
| Soccer | 4 | 57.1 | 0 | 0 | 0 |
| Tennis/Squash | 3 | 42.9 | 0 | 0 | 0 |
| Ski | 1 | 14.3 | 0 | 0 | 0 |

| ^a^ Breakdown of the sports by various subgroups as performed prior to index ACL injury and after successful treatment of septic arthritis  ^b^ Age at the time of first treatment of septic arthritis at our institution  ^c^ Patients with initial graft removal and with (+) or without (-) revision ACLR by the time of follow up  ACLR, anterior cruciate ligament reconstruction. |
| --- |
